# Effects of Cholinesterase Inhibitor Medication on QTc Interval in Memory Clinic Patients

**DOI:** 10.1177/10600280251328530

**Published:** 2025-05-06

**Authors:** Hanna Karita Isotalo, Joanna Karoliina Lehtovaara, Laura Linnea Ekblad, Maria Susanna Nuotio, Ville Lauri Johannes Langén

**Affiliations:** 1 Department of Geriatric Medicine, Faculty of Medicine, Turku University Hospital, University of Turku, Turku, Finland; 2 Southwest Finland Wellbeing Services County, Turku, Finland; 3 Faculty of Medicine, University of Turku, Turku, Finland; 4 Turku PET Centre, Turku University Hospital, University of Turku, Turku, Finland; 5 Division of Medicine, Turku University Hospital, University of Turku, Turku, Finland

**Keywords:** cholinergics, Alzheimer disease, geriatrics, drug-related problems, drug safety

## Abstract

**Background:**

Cholinesterase inhibitors (ChEIs)—donepezil, rivastigmine, and galantamine—are beneficial in treating Alzheimer disease (AD). However, due to their impact on extra-cerebral acetylcholine signaling, concerns about cardiac adverse effects, including QT interval prolongation, persist. Despite this, evidence-based guidelines for electrocardiogram (ECG) monitoring during ChEI treatment are lacking, and prior studies on ChEIs and corrected QT intervals (QTc) yield inconsistent findings.

**Objective:**

This study aimed to investigate the association between ChEI use and changes in QTc intervals among older adults.

**Methods:**

We collected retrospective data from first-time visitors to the geriatric memory clinic of Turku City Hospital in 2017 and 2019. We included patients who were newly prescribed ChEIs and had ECG data available (n = 126, mean age 81.1 years, 56.3% female). QTc prolongation was defined as ≥460 ms in females and ≥450 ms in men. Paired *t* tests compared QTc means before and during ChEI use, and McNemar tests analyzed changes in the proportion of prolonged QTc.

**Results:**

Mean ± SD QTc (ms) before versus during ChEI use was: 420.8 ± 24.0 versus 423.9 ± 28.0 (*P* = .13) for donepezil; 416.0 ± 20.4 versus 416.5 ± 26.1 (*P* = .92) for galantamine; 416.1 ± 22.3 versus 409.6 ± 20.1 (*P* = .30) for rivastigmine; and 419.7 ± 23.4 versus 421.5 ± 27.3 (*P* = .34) for all ChEIs. Prolonged QTc occurred in 7.9% of patients before versus 12.7% during ChEI use (*P* = .21).

**Conclusion and Relevance:**

We found no statistically significant association between ChEI use and QTc interval prolongation or an increased proportion of pathological QTc values during ChEI treatment. Larger studies are warranted to establish evidence-based recommendations on ECG monitoring during ChEI medication.

## Introduction

Dementia is a major cause of disability, dependency, and mortality worldwide, with Alzheimer disease (AD) accounting for 60% to 70% of dementia cases.^1^ Although a cure for AD still remains elusive, progression of cognitive symptoms may be delayed with cholinesterase inhibitors (ChEIs) including donepezil, rivastigmine, and galantamine, and the N-methyl-aspartate (NMDA) receptor antagonist memantine.^2^ ChEIs are considered the first-line therapy for mild-to-moderate AD, while memantine is recommended for moderate-to-severe stages.^3^ Recently, the U.S. Food and Drug Administration (FDA) approved antiamyloid drugs lecanemab, aducanumab, and donanemab for AD treatment, although aducanumab was later discontinued by the manufacturer.^4,5^ However, the widespread use of these antiamyloid therapies may be hindered due to their high cost, remaining questions concerning their safety and effectiveness, and limited availability outside the United States. As a result, ChEIs still play an important role in the treatment of AD, but there is a significant lack of clinical guidelines on how to balance their potential benefits against the risks of adverse effects.

In previous literature, ChEIs have been associated with adverse effects on the gastrointestinal, neurological, and cardiovascular systems.^6^ The possible cardiac adverse effects include bradycardia, syncope, QT prolongation, and Torsades de Pointes (TdP) ventricular tachycardia.^7^ However, the full extent of the impact of ChEIs on QT interval remains unclear. Research on this topic has primarily consisted of either case reports of QT prolongation and TdP^8-15^ or small observational studies with conflicting reports regarding the association of ChEI use with corrected QT (QTc) interval.^16-18^ Current clinical guidelines, including those from major organizations, do not specifically address the issue of QT prolongation with ChEI use.^19-21^ The Finnish guideline does suggest ECG monitoring for patients taking donepezil due to possible QT interval prolongation with this drug, but the potential cardiac risks associated with other ChEIs are not addressed.^22^

To address this gap in the existing literature, we conducted a study using data obtained from a specialized memory clinic. We collected electrocardiogram (ECG) readings before and after patients were prescribed ChEIs to explore their potential impact on QT interval. Our underlying hypothesis was that the use of ChEIs may prolong QT interval, which could ultimately increase the risk of cardiac arrhythmias and sudden cardiac death in ChEI-treated patients.

## Materials and Methods

### Study Design

The SafeALZ study is a research initiative where we collected retrospective register-based data on first-time visitors to the geriatric memory clinic of Turku City Hospital in 2017 and 2019. The gathered information included demographic details (age, sex), details on other major diseases, current laboratory results, Consortium to Establish a Registry for Alzheimer’s Disease (CERAD) and/or Mini-Mental State Examination (MMSE) test results, initiation date of ChEI and/or memantine, and ECG data before and during ChEI or memantine.

### Study Inclusion-Exclusion Criteria

**Figure 1.**
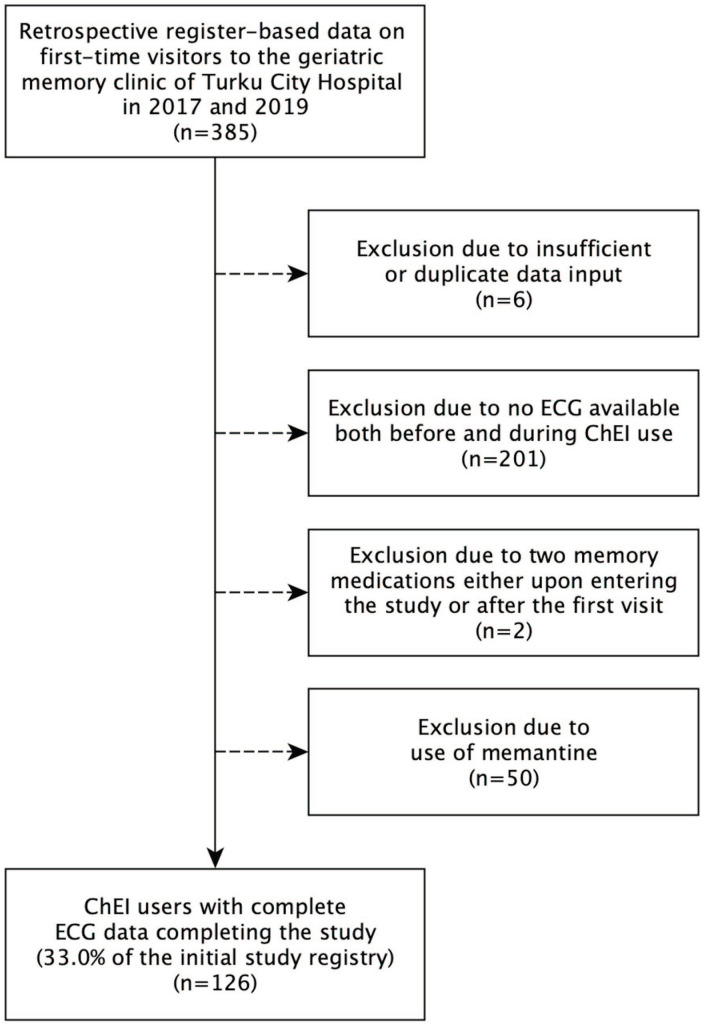
Flowchart of SafeALZ study inclusion-exclusion criteria.

In this study, we included patients who were newly prescribed ChEI medication and had standard 12-lead ECG data available both before and during ChEI use. The detailed exclusion criteria are shown in Figure 1.

### ECG Measurements

We manually read all ECGs for QT interval length following the method described by Isbister et al.^23^ We performed QT interval measurements in 3 limb leads (I, II, and aVF or aVL) and 3 chest leads (V2, V4, and V5 or V6) using a clear measuring ruler. We measured the QT interval from the onset of the QRS complex to the end of the T wave. We identified the end of the T wave by a visual method, determining the point where the T-wave returns to baseline (isoelectric line). The median QT of the individual leads was then calculated. We obtained the heart rate (HR) from the automated readout of the ECG machine. In 45.2% of the ECGs, we could not reliably measure the QT interval in all 6 leads due to poor signal quality or low voltage amplitudes. In those cases, we opted to use the available lead data to compute the median QTs.

We chose manual QT interval measurements over automated methods because prior studies have shown that manual measurement provides greater accuracy, particularly in cases of low-amplitude signals or abnormal T-wave morphologies where automated algorithms may misidentify the QT endpoint. By incorporating multiple leads and calculating the median QT, this method reduces the impact of outlier measurements, thereby improving overall accuracy.^23^ In addition, manual measurement has demonstrated good interrater reliability when performed using a standardized approach.^24^

### Heart Rate Correction of the QT Interval

We calculated the QTc interval using Bazett correction formula (QTc = QT/√(60/HR)), the most commonly employed correction method in clinical practice. Prolonged QTc was defined as ≥460 ms in females and ≥450 ms in men, according to guidelines for the standardization and interpretation of ECGs.^25^

### Statistical Analyses

We conducted statistical analyses using the R statistical software, version 4.2.2 (R Core Team, Vienna, Austria), with a significance level set at .05. Comparing ECGs before and during ChEI medication, we assessed differences in QTc values with the 2-tailed paired *t* test and proportions of patients with prolonged QTc with the McNemar test.

### Sensitivity Analyses

As a sensitivity analysis, we repeated all analyses excluding patients without data of QT interval measurements across all 6 leads (I, II, aVF/aVL, V2, V4, V5/V6).

### Ethics

The participants of the SafeALZ study were not contacted. Therefore, according to Finnish legislation, neither ethics committee approval nor informed consent was required. The study adhered to the tenets of the Declaration of Helsinki.

## Results

### Patient Characteristics

The baseline characteristics of the study population are listed in Table 1. A total of 126 patients (56.3% female) met the inclusion-exclusion criteria of this study. The mean age ± standard deviation (SD) was 81.1 ± 4.1 years. Seventy-seven percent of the patients were treated with donepezil, 11.9% with galantamine, and 11.1% with rivastigmine. The mean times ± SD from the first ECG to ChEI initiation and from ChEI initiation to the second ECG were 146.5 ± 164.6 and 144.2 ± 222.8 days, respectively.

**Table 1.** Characteristics of Patients Treated With ChEI Medication in Memory Clinic.

Variable	All patients (N = 126)
Age, years	81.1 (4.1) (range: 70-91.9)
Female	71 (56.3%)
Mini-Mental State Examination	22.8 (3.3) (range: 12-29)
Hemoglobin (g/L)	139.6 (14.4)
Serum creatinine (µmol/L)	85.4 (25.7)
eGFR (mL/min/1.73m²)	66.9 (13.6)
Serum potassium (mmol/L)	4.0 (0.3)
Serum sodium (mmol/L)	141.3 (3.3)
Serum ionized calcium (mmol/L)	1.2 (0.1)
BMI (kg/m²)	25.5 (4.4)
ChEI medication	
Donepezil (oral)	97 (77.0%)
Galantamine (oral)	15 (11.9%)
Rivastigmine (oral)	1 (0.8%)
Rivastigmine (transdermal)	13 (10.3%)
Time from first ECG to initiation of ChEI, days	146.5 (164.6)
Time from ChEI initiation to second ECG, days	144.2 (222.8)
QTc interval before ChEI, ms	419.7 (23.4)
QTc interval after initiation of ChEI, ms	421.5 (27.3)
Pathological QTc before ChEI	10 (7.9%)
Pathological QTc during ChEI	16 (12.7%)

Values are means (and standard deviations) for continuous data and numbers (and percentages) for categorical data, unless otherwise noted. Pathological QTc defined as ≥450 ms for male and ≥460 ms for female participants.

Laboratory results prior to ChEI medication initiation were available for 95.2% of patients (hemoglobin), 93.7% (serum creatinine and eGFR), 92.1% (serum potassium and sodium), and 58.7% (serum ionized calcium).

Abbreviations: BMI, body mass index; ChEI, cholinesterase inhibitor; ECG, electrocardiogram; eGFR, estimated glomerular filtration rate; QTc, corrected QT interval.

### QTc Intervals Before Versus During ChEI Medication

**Figure 2.**
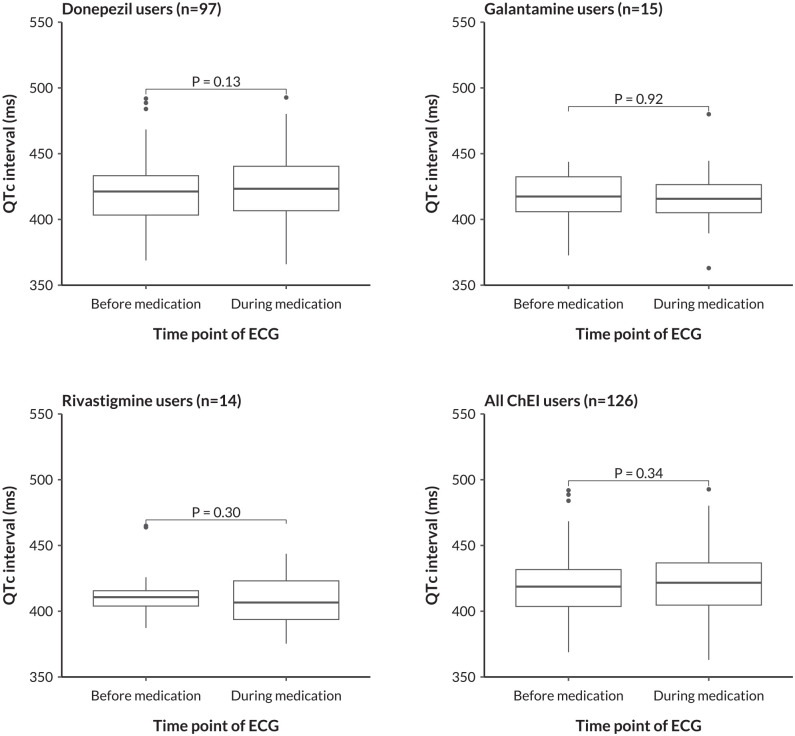
Differences in QTc interval as measured before versus during ChEI medication. The depicted *P* values were obtained from paired *t* test models. ChEI, cholinesterase inhibitor; QTc, corrected QT interval; ECG, electrocardiogram.

Table 2 and Figure 2 provide details on the differences in QTc interval before versus during ChEI medication. We analyzed these differences for each ChEI individually and for all ChEIs combined. Mean values ± SDs of QTc intervals (ms) measured before versus during ChEI medication were 420.8 ± 24.0 versus 423.9 ± 28.0 (*P* = .13) in donepezil users, 416.0 ± 20.4 versus 416.5 ± 26.1 (*P* = .92) in galantamine users, 416.1 ± 22.3 versus 409.6 ± 20.1 (*P* = .30) in rivastigmine users, and 419.7 ± 23.4 versus 421.5 ± 27.3 (*P* = .34) in all ChEI users combined.

**Table 2.** QTc Values Measured Before and During ChEI Medication.

		QTc before medication		QTc during medication				
Group	n	Mean	*SD*	Mean	*SD*	Mean difference	t	*P* value
Donepezil	97	420.8	24.0	423.9	28.0	−3.2	−1.53	0.13
Galantamine	15	416.0	20.4	416.5	26.1	−0.6	−0.10	0.92
Rivastigmine	14	416.1	22.3	409.6	20.1	6.4	1.08	0.30
**Overall**								
All ChEIs	126	419.7	23.4	421.5	27.3	−1.8	−0.96	0.34

Abbreviations: ChEI, cholinesterase inhibitor; QTc, corrected QT interval.

### Proportion of Pathological QTc Intervals Before Versus During ChEI Medication

**Figure 3.**
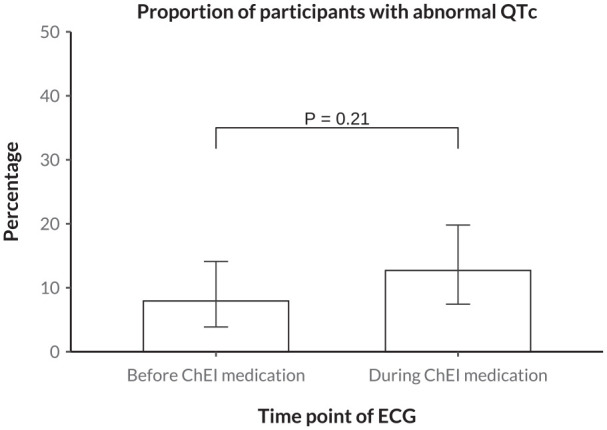
Difference in the proportion of pathological QTc intervals before versus during ChEI medication. The depicted *P* value was obtained from a McNemar test model. Pathological QTc interval was defined as ≥450 ms in women and ≥460 ms in men. QTc, corrected QT interval; ChEI, cholinesterase inhibitor; ECG, electrocardiogram.

**Figure 4.**
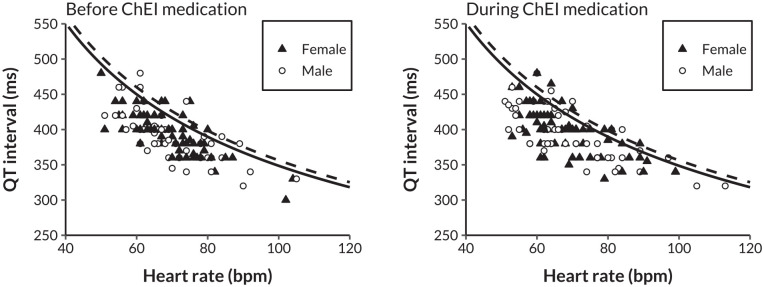
Scatter plots of QT interval by heart rate before and during ChEI medication. The solid line represents the threshold for a pathological corrected QT interval in males (≥450 ms), while the dashed line indicates the threshold for females (≥460 ms). ChEI, cholinesterase inhibitor; bpm, beats per minute; ms, milliseconds.

Figure 3 shows the number of patients with a pathological QTc interval before and during ChEI medication. Before ChEI, 10 patients (7.9%) had a pathological QTc interval, compared with 16 patients (12.7%) during ChEI (*P* = .21 for difference). Figure 4 displays the relationship between the QT interval and heart rate before and during ChEI medication use, with thresholds for pathological QT intervals indicated separately for males and females. The scatter plots suggested only negligible changes in the prevalence of QT intervals exceeding these pathological thresholds following the initiation of ChEI therapy. In addition, the distribution of QT intervals in relation to heart rate remained largely consistent before and during treatment.

### Sensitivity Analyses

We reiterated all our analyses using data only from patients who had manual QT measurements for all 6 leads (n = 69). The baseline characteristics of the sensitivity analysis study population are provided in Supplementary Table S1. Compared with the original analyses, the mean age of the patients remained largely unchanged (81.1 ± 4.1 vs 80.9 ± 4.2 years). A slightly higher proportion of patients were female (56.3% vs 60.9%). Among these patients, 76.8% were treated with donepezil, 11.6% with galantamine, and 11.6% with transdermal rivastigmine. There were no patients receiving oral rivastigmine.

Means ± SDs of QTc intervals (ms) measured before versus during ChEI medication were 415.7 ± 19.7 versus 420.1 ± 28.8 (*P* = .11) in donepezil users, 404.3 ± 19.6 versus 408.9 ± 11.0 (*P* = .51) in galantamine users, 406.9 ± 9.1 versus 402.8 ± 15.7 (*P* = .50) in rivastigmine users, and 413.4 ± 19.0 versus 416.8 ± 26.6 (*P* = .14) in all ChEI users combined (Supplementary material, Table S2, Figure S1). Before ChEI, 1 patient (1.4%) had a pathological QTc interval, compared with 7 patients (10.1%) during ChEI (*P* = .04 for difference) (Supplementary material, Figure S2).

Supplementary Figure S3 illustrates the relationship between the QT interval and heart rate before and during ChEI medication use in a subpopulation comprising only patients with manual QT measurements available for all 6 leads. The results appeared mainly in accordance with those observed in the main population, though the prevalence of QT intervals exceeding the threshold for pathological QT intervals following the initiation of ChEI therapy seemed slightly more prominent in these sensitivity analyses.

## Discussion

The results of our study showed that there were no statistically significant differences in QTc intervals measured before ChEI medication compared with those measured during ChEI medication. This pattern was consistent across all ChEIs examined: donepezil, galantamine, and rivastigmine. Moreover, the initiation of ChEI medication did not associate with a statistically significant increase in the proportion of pathological QTc intervals.

In sensitivity analyses conducted exclusively with patients who had manual QT measurements for all 6 leads, the results remained largely unchanged. However, the proportion of patients with pathological QTc before versus during ChEI treatment increased to a statistically significant extent. Given that this was not our primary analysis, involved a smaller subset of the study population, and may be influenced by the problem of multiple comparisons, some cautiousness is warranted in interpreting these results.

Our findings contribute to and mostly align with the preexisting scarce literature on this topic. Consistent with our results, Bordier et al found no significant differences in QTc values measured at baseline compared with those recorded at 1, 2, and 8 months after the initiation of donepezil therapy in patients with AD. In addition to ECG measurements, they also monitored blood pressure and heart rate and categorized the study population based on the use of medications known to slow cardiac conduction and/or heart rate. However, their study had several limitations: it focused exclusively on donepezil, did not specify whether QTc intervals were calculated manually or automatically, and had a relatively small sample size, with only 22 patients completing the 8-month follow-up.^17^ Isik et al^16^ also reported congruent results in their study with largely similar methods. The strength of that study was its slightly larger sample size (n = 162), but they relied on automatic determination of QTc intervals with an ECG apparatus, which can be considered a limitation. In alignment with our findings, in 4 double-blinded, multicenter, placebo-controlled phase III clinical trials (n = 2756), treatment with rivastigmine was not associated with any adverse effects on ECG parameters, including QTc intervals.^26^ In a large (n = 73 475) retrospective cohort study comparing cardiovascular effects of ChEI monotherapy, memantine monotherapy, and combination therapy with ChEI and memantine, no statistically significant association with ChEI use and QTc prolongation was observed. Notably, QTc prolongation was identified solely through reported International Classification of Diseases, Ninth Revision (ICD-9) codes, rather than by direct measurement of the QTc intervals.^27^

Kuwahata et al^18^ reported divergent results compared with our study. They not only compared QTc intervals before and after the initiation of donepezil but also investigated this question in a case-control setting. Their study, which was of a comparable size with ours, found that QTc interval was significantly longer during the donepezil medication than at baseline or in an age- and sex-matched control group without donepezil use. Furthermore, the percentage of patients with QTc prolongation was significantly higher in the donepezil than in the control group. The additional analyses in the case-control setting are a clear strength of that study, while their employing an automatic QTc measurement approach can be regarded as a limitation. The dissimilarity of the results of Kuwahata et al underscores the potential impact of methodological variations, particularly in the mode of QTc measurement. This emphasizes the necessity for standardization in QTc assessment methodologies to enhance the reliability and comparability of findings in the realm of ChEI-related cardiac effects. In addition, while the authors examined the use of concomitant medications with potential QT-prolonging effects in both groups and found no significant differences, their analysis was limited to histamine H_2_ receptor blockers and loop or thiazide diuretics. This represents a significant limitation, given the extensive range of QT-prolonging drugs cataloged in resources like CredibleMeds. Without a detailed evaluation of specific medications and their varying levels of risk, it remains challenging to determine whether such drugs confounded the observed association between donepezil and QTc prolongation.^18^ Similarly, we acknowledge that our study did not include information on concomitant medications that could prolong the QT interval, representing a shared limitation that warrants consideration in the interpretation of our findings.

In a previous single-center retrospective analysis by Kho et al^28^ involving 59 patients using donepezil, the authors reported significant prolongation of PR, QRS, and QT intervals associated with donepezil use. A notable strength of their study was the inclusion of data on comorbidities, concurrent medications with potential cardiac effects, and the dose and duration of donepezil therapy. They also manually measured the QT interval across multiple leads, calculated the median QT, and applied all 4 commonly used formulae to correct QT intervals for heart rate, as well as the QT nomogram to assess arrhythmogenic risk. Although their findings differed from ours, it is important to note that their ECG recordings during donepezil use were obtained during acute hospital admissions. This setting may have introduced confounding factors that could have influenced the observed QT interval prolongation.^28^

Delay in ventricular repolarization may lead to possibly life-threatening TdP ventricular tachycardia. A data mining study of the FDA Adverse Event Reporting System revealed an elevated reporting odds of TdP associated with donepezil, with 11 cases reported.^29^ In a nationwide Swedish register-based cohort study, 7 cases of TdP were found among donepezil users, with a notable 71% of them concurrently using other TdP-classified drugs.^30^ In 2015, donepezil was classified in the CredibleMeds “known risk” category for medications with a documented association with acquired long-QT syndrome (ALQTS) and TdP. Rivastigmine is listed as having a “possible risk” of TdP, while galantamine is categorized under “conditional risk” of TdP.^31^ In a recent pharmacovigilance study using the FAERS database, Zhang et al^32^ investigated the association between ChEIs and the development of TdP and QT interval prolongation by analyzing adverse event reports to identify cases linked to ChEIs. They identified a total of 557 cases of TdP/QT prolongation associated with ChEIs. The patients were predominantly 65 years or older, with a significantly higher proportion of females. Donepezil exhibited the strongest signal detected by disproportionality analysis. The median onset intervals for TdP/QT prolongation associated with ChEIs were 52 days (IQR 5-539) for donepezil, 48.5 days (IQR 7-695.75) for galantamine, and 82 days (IQR 8-420.75) for rivastigmine. These findings highlight considerable variability in the timing of TdP/QT prolongation onset following the initiation of ChEI therapy, which could suggest a potential need for repeated ECG monitoring in certain clinical settings. However, it is important to note that among the top 30 drugs frequently used in combination with ChEIs were many known QT-prolonging agents, such as citalopram. Also, signal detection merely suggests a potential risk but does not confirm a definitive causal relationship. To better compare the QT prolongation potential between different ChEIs, more studies with a design similar to ours would be needed. Among such studies, which have been referenced above, 3 assessed only donepezil,^17,18,33^ and only 1 addressed all ChEIs, demonstrating no cardiac adverse effects for any ChEI.^16^ We argue that more robust evidence is needed to determine whether different ChEIs warrant a different approach in estimating their cardiac adverse effect potential.

In contrast to the registry and database studies mentioned in the preceding paragraph, a review of 61 788 reported adverse drug reactions in the Swedish pharmacovigilance database identified no cases of TdP associated with ChEI use.^34^ Also, in an analysis of the Australian Drug Reaction Advisory Committee (ADRAC) reports, no cases of TdP associated with ChEI use were found.^13^ A recent systematic review with meta-analysis of 60 randomized controlled trials (n = 12 463) found no association between donepezil and TdP, death, ventricular tachycardia, ventricular fibrillation and flutter, syncope, and seizures, but only 33 of 60 trials conducted ECG monitoring.^35^ These conflicting results regarding the TdP data underline the need for further research on this field.

Quality research on the cardiac safety of ChEIs is crucial for the development of national and international guidelines regarding ECG-guided monitoring during treatment. In Finland, clinicians benefit from existing national guidelines, called the Current Care Guidelines for Dementia, which provide comprehensive recommendations for the treatment of patients with cognitive impairment.^22^ However, within these Current Care Guidelines, while there is promotion of ECG monitoring for ChEI-induced cardiac effects and acknowledgment of the potential for QT prolongation by donepezil, detailed instructions regarding monitoring protocols and explicit acknowledgment of cutoff values for discontinuing the medication are absent. This limitation is likely due to the current lack of conclusive research in this area.

The gaps in standardized guidelines for ECG interpretation in the context of ChEI treatment are reflected in clinical practice, as evidenced by our recent research on this topic. Our previous study investigated the challenges faced by clinicians in interpreting ECG findings when prescribing medications for cognitive impairment. A survey of health care providers revealed significant variability in the interpretation of ECG changes deemed contraindications for initiating or escalating ChEI therapy.^36^

In addition to national guidelines, Finnish clinicians benefit from access to valuable drug safety resources such as the INXBASE drug-drug interaction database^37^ and its complementary database, RISKBASE,^38^ which provides a comprehensive risk profile of patient’s medication. Presently, the latter database categorizes the potential risk of medication-induced QT prolongation for donepezil as class B, indicating a “mildly increased risk.” In contrast, galantamine and rivastigmine are categorized as class A, denoting “no known increased risk.” Databases like these in Finland and elsewhere would benefit from additional large-scale research on the cardiac safety of the use of various ChEIs.

The strength of our study lies in our methodology, wherein we opted to use the median of multiple manually measured QT intervals in order to avoid potential inaccuracies associated with automated measurements.^23,39^

An important limitation of our study is the omission of the potential influence of other QT-prolonging medications, electrolyte imbalances, and comorbidities. We did not report comorbidity data or use of other medications due to the retrospective nature of the medical records, which may introduce bias and fail to fully capture patients’ medical histories or medication use.

Selection bias may also be a concern, as patients with prolonged QT intervals might have been more likely to receive memantine instead of ChEI therapy. This clinical decision-making, influenced by the Finnish medical databases, may have diluted our findings. Clinicians in memory clinic are likely aware of the potential cardiac adverse effects of ChEIs and might therefore avoid prescribing these medications to patients at higher risk for QT prolongation, such as those already taking QT-prolonging drugs, with marked bradycardia, or with preexisting QT prolongation.

We also lacked data on ChEI medication purchases. However, in dementia care settings, caregivers or home care providers typically manage medication administration, and adherence to the prescribed medications was monitored during follow-up calls made by memory clinic nurses. While this monitoring suggests reasonable adherence, the lack of precise purchase data remains a limitation. In addition, it is important to acknowledge that patients have the right to refuse medication, and medications may also be intermittently held, which could further impact adherence.

Another significant limitation of our study is its retrospective design, which prevented us from predefining the timing of ECGs. Instead, we relied on identifying the closest ECG recorded before and after medication initiation. On the other hand, the literature provides limited guidance on the specific timeframe for the onset of possible QT prolongation after ChEI initiation. The risk and timing of QT prolongation may vary based on factors such as comorbidities, electrolyte disturbances, and concurrent medications that affect the QT interval or heart rate. In the study by Bordier et al,^17^ which found no statistically significant QTc interval prolongation, ECGs were performed at baseline and at 1, 2, and 8 months following donepezil initiation. By comparison, the pharmacovigilance study by Zhang et al^32^ observed considerable variability in the timing of TdP/QT prolongation onset after ChEI therapy initiation.

Moreover, the inclusion of age- and sex-matched controls would have strengthened the study and allowed for more robust comparisons. Unfortunately, the retrospective nature of our data collection did not permit this, limiting our ability to control for potential confounding variables related to demographic differences. In addition, our study is limited by the small sample size of patients using galantamine and rivastigmine, with the majority of patients treated with donepezil. This imbalance may reduce the generalizability of findings across all ChEI medications.

It is also important to note that the previous case reports and studies may not be fully comparable with our study population, as differences in patient demographics, comorbidities, and concurrent medication use may exist. These factors could differ significantly from our cohort, which comprised older adults aged 70 and above receiving routine care at a memory clinic. Such variations in study populations may partly explain the discrepancies in QTc changes observed across previous studies. Furthermore, our study was conducted in a predominantly white population, as Finland is relatively racially and ethnically homogenous among older generations. This may limit the generalizability of our findings to more diverse populations.

## Conclusion and Relevance

In conclusion, our retrospective register-based study did not identify any statistically significant association between ChEI medication and QT interval prolongation. However, in a sensitivity analysis, which included data only from patients with manual QT measurements available for all 6 leads, there was a statistically significant increase in the proportion of patients with pathological QTc when comparing the period before and during ChEI treatment. This finding warrants careful interpretation, given that it derives from a smaller subset of the study population and is distinct from our main analysis, with potential susceptibility to multiplicity effects.

This study contributes to the limited evidence regarding the potential cardiac adverse effects of ChEIs by analyzing real-world data from an outpatient memory clinic population of older adults aged 70 and above. Although we acknowledge that information on comorbidities and concomitant medications would have been valuable to display, our study population reflects typical clinical practice, as patients were not excluded based on the presence of possible comorbidities or concurrent medication use.

Further studies with larger cohorts are warranted to deepen our understanding of the potential impact of ChEI medication on QT prolongation, particularly in vulnerable geriatric populations. Such research initiatives will also be essential for establishing comprehensive, evidence-based guidelines for ECG monitoring during ChEI therapy. Given that older patients are more likely to present with multiple risk factors for QT interval prolongation and TdP—including advanced age, a history of heart disease, electrolyte imbalances, and concurrent use of QT-prolonging drugs—it is advisable to exercise caution.^34^ Each patient’s individual risk factors should be carefully assessed when prescribing ChEI medications, with ECG monitoring implemented if necessary.

## Supplemental Material

sj-docx-1-aop-10.1177_10600280251328530 – Supplemental material for Effects of Cholinesterase Inhibitor Medication on QTc Interval in Memory Clinic Patients Supplemental material, sj-docx-1-aop-10.1177_10600280251328530 for Effects of Cholinesterase Inhibitor Medication on QTc Interval in Memory Clinic Patients by Hanna Karita Isotalo, Joanna Karoliina Lehtovaara, Laura Linnea Ekblad, Maria Susanna Nuotio and Ville Lauri Johannes Langén in Annals of Pharmacotherapy
