# Supplementary material for: Effects of Cholinesterase Inhibitor Medication on QTc Interval in Memory Clinic Patients
Source: Ann Pharmacother. 2025 May 6;60(1):42–52. doi: 10.1177/10600280251328530 (PMC12696191; doi:10.1177/10600280251328530)
Supplement: sj-docx-1-aop-10.1177_10600280251328530 – Supplemental material for Effects of Cholinesterase Inhibitor Medication on QTc Interval in Memory Clinic Patients [file sj-docx-1-aop-10.1177_10600280251328530.docx]

**Supplementary material**

**Table S1.** Characteristics of patients treated with ChEI medication in memory clinic in a subpopulation comprising only those with manual QT measurements available for all six leads.

**Table S2.** QTc values measured before and during ChEI medication in a subpopulation comprising only patients with manual QT measurements available for all six leads.

**Figure S1.** Differences in QTc intervals measured before and during ChEI medication in a subpopulation comprising only patients with manual QT measurements available for all six leads.

**Figure S2.** Difference in the proportion of pathological QTc intervals before and during ChEI medication in a subpopulation comprising only patients with manual QT measurements available for all six leads.

**Figure S3.** Scatter plots of QT interval by heart rate before and during ChEI medication in a subpopulation comprising only patients with manual QT measurements available for all six leads.

| **Table S1. Characteristics of patients treated with ChEI medication in memory clinic in a subpopulation comprising only those with manual QT measurements available for all six leads** | |
| --- | --- |
| **Variable** | **All patients (N = 69)** |
| Age, years | 80.9 (4.2) |
| Female | 42 (60.9%) |
| Mini-Mental State Examination | 22.6 (3.0) (range: 14–29) |
| Hemoglobin (g/L) | 140.1 (13.1) |
| Serum creatinine (µmol/L) | 84.2 (27.9) |
| eGFR (mL/min/1.73m²) | 68.1 (12.5) |
| Serum potassium (mmol/L) | 4.0 (0.3) |
| Serum sodium (mmol/L) | 141.3 (3.3) |
| Serum ionized calcium (mmol/L) | 1.2 (0.1) |
| BMI (kg/m²) | 25.8 (4.3) |
| ChEI medication |  |
| Donepezil (oral) | 53 (76.8%) |
| Galantamine (oral) | 8 (11.6%) |
| Rivastigmine (transdermal) | 8 (11.6%) |
| Time from 1st ECG to initiation of ChEI, days | 133.4 (152.8) |
| Time from ChEI initiation to 2nd ECG, days | 128.4 (194.9) |
| QTc interval before ChEI, ms | 413.4 (19.0) |
| QTc interval after initiation of ChEI, ms | 416.8 (26.6) |
| Pathological QTc before ChEI | 1 (1.4%) |
| Pathological QTc during ChEI | 7 (10.1%) |
| Values are means (and standard deviations) for continuous data and numbers (and percentages) for categorical data, unless otherwise noted. Pathological QTc defined as ≥450 ms for male and ≥460 ms for female participants. | |
| Laboratory results prior to ChEI medication initiation were available for 95.6% of patients for hemoglobin, 92.8% for serum creatinine and eGFR, 89.9% for serum potassium and sodium, and 62.3% for serum ionized calcium. | |
| BMI, body mass index; ChEI, cholinesterase inhibitor; ECG, electrocardiogram; eGFR, estimated glomerular filtration rate; QTc, corrected QT interval. | |

| **Table S2. QTc values measured before and during ChEI medication in a subpopulation comprising only patients with manual QT measurements available for all six leads** | | | | | | | | | |
| --- | --- | --- | --- | --- | --- | --- | --- | --- | --- |
|  | | **QTc before medication** | |  | **QTc during medication** | |  | | |
| **Group** | ***n*** | **Mean** | ***SD*** |  | **Mean** | ***SD*** | **Mean difference** | ***t*** | ***p*-value** |
| Donepezil | 53 | 415.7 | 19.7 |  | 420.1 | 28.8 | -4.4 | -1.63 | 0.11 |
| Galantamine | 8 | 404.3 | 19.6 |  | 408.9 | 11.0 | -4.5 | -0.69 | 0.51 |
| Rivastigmine | 8 | 406.9 | 9.1 |  | 402.8 | 15.7 | 4.1 | 0.71 | 0.50 |
| **Overall** |  |  |  |  |  |  |  |  |  |
| All ChEIs | 69 | 413.4 | 19.0 |  | 416.8 | 26.6 | -3.4 | -1.49 | 0.14 |
| QTc, corrected QT interval; ChEI, cholinesterase inhibitor. | | | | | | | | | |


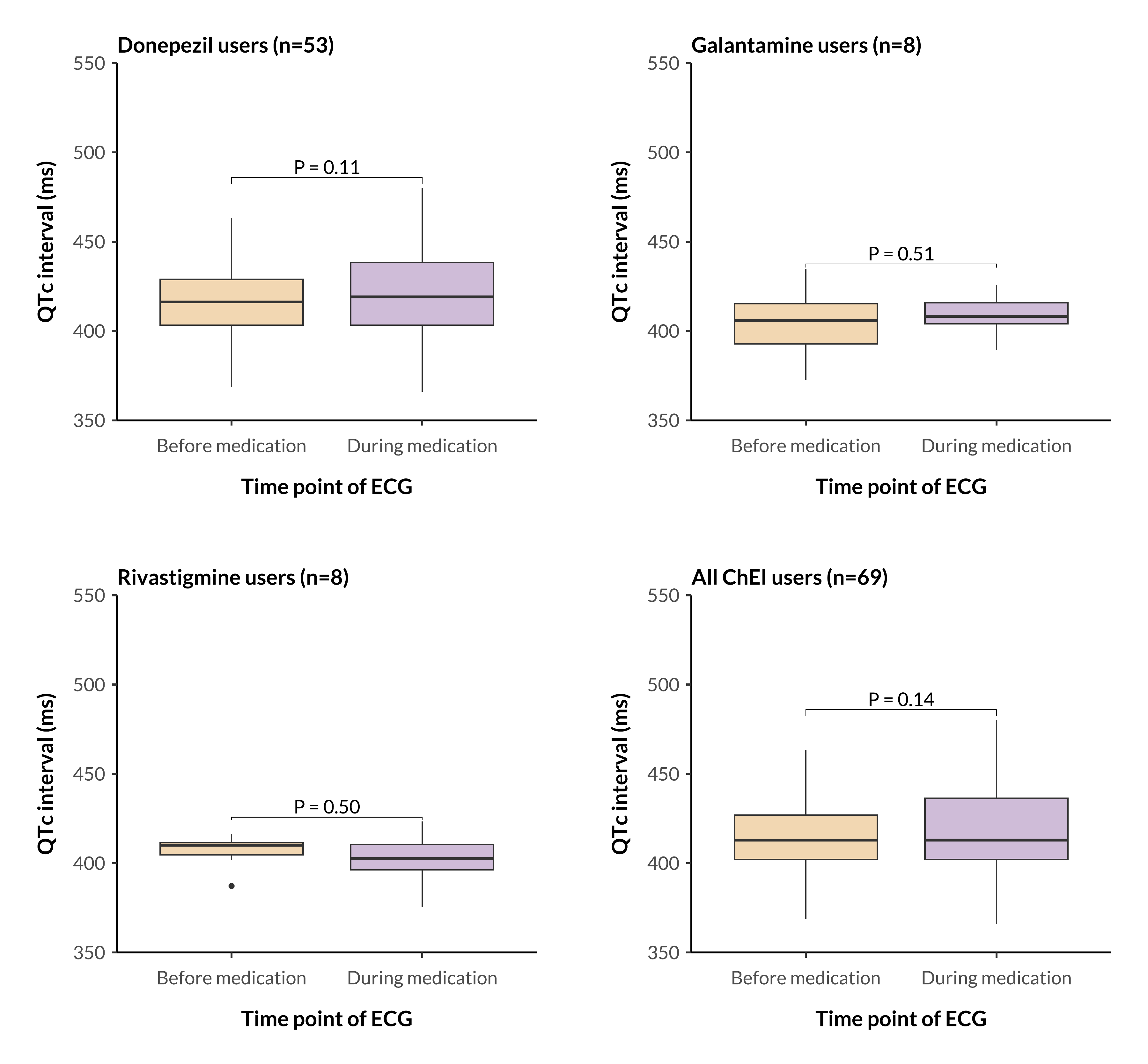


**Figure S1.** Differences in QTc intervals measured before and during ChEI medication in a subpopulation comprising only patients with manual QT measurements available for all six leads. The depicted P-values were obtained from paired t-test models. ChEI, cholinesterase inhibitor; QTc, corrected QT interval; ECG, electrocardiogram


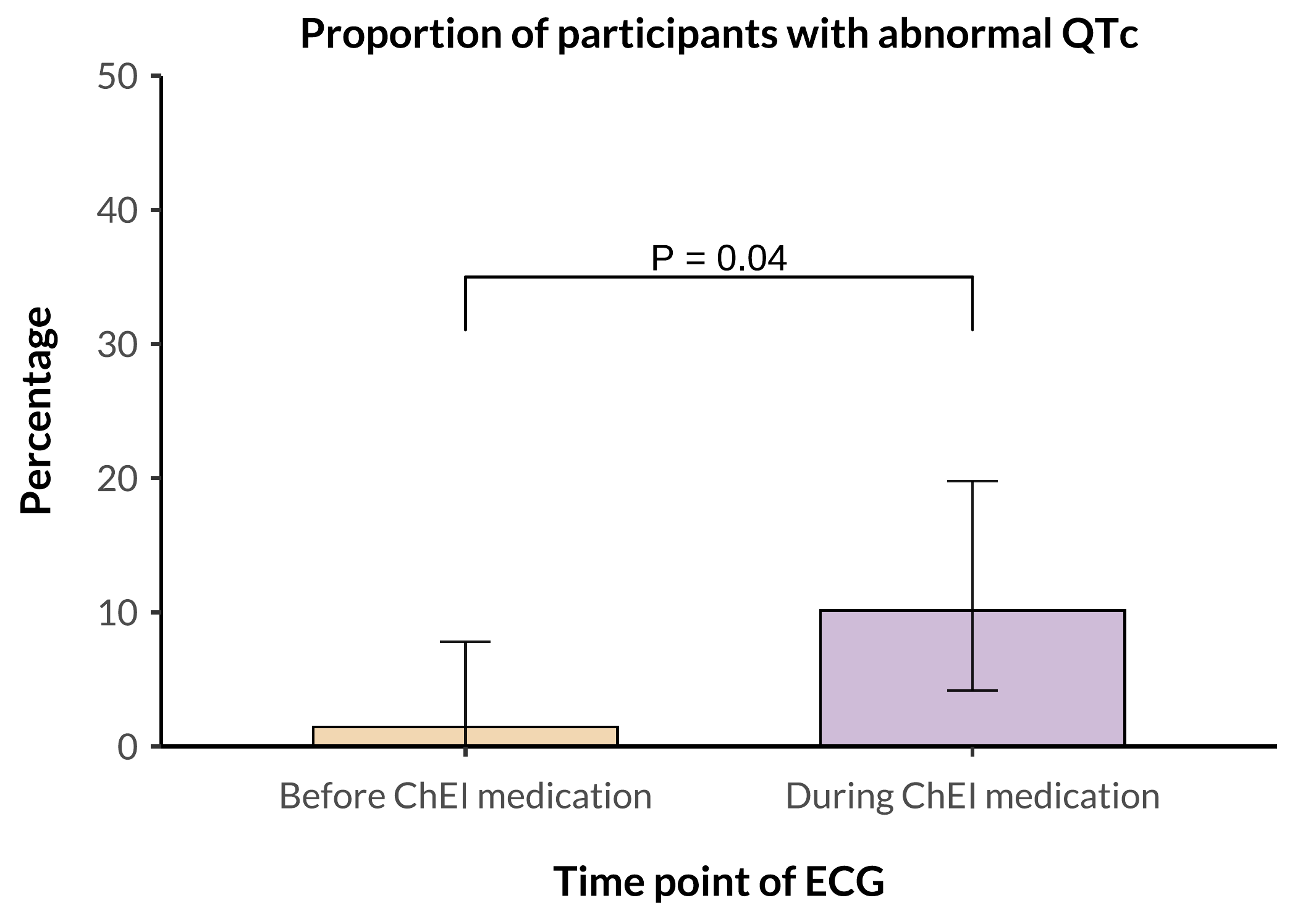


**Figure S2.** Difference in the proportion of pathological QTc intervals before and during ChEI medication in a subpopulation comprising only patients with manual QT measurements available for all six leads. The depicted P-value was obtained from a McNemar's test model. Pathological QTc interval was defined as ≥450 ms in women and ≥460 ms in men. QTc, corrected QT interval; ChEI, cholinesterase inhibitor; ECG, electrocardiogram


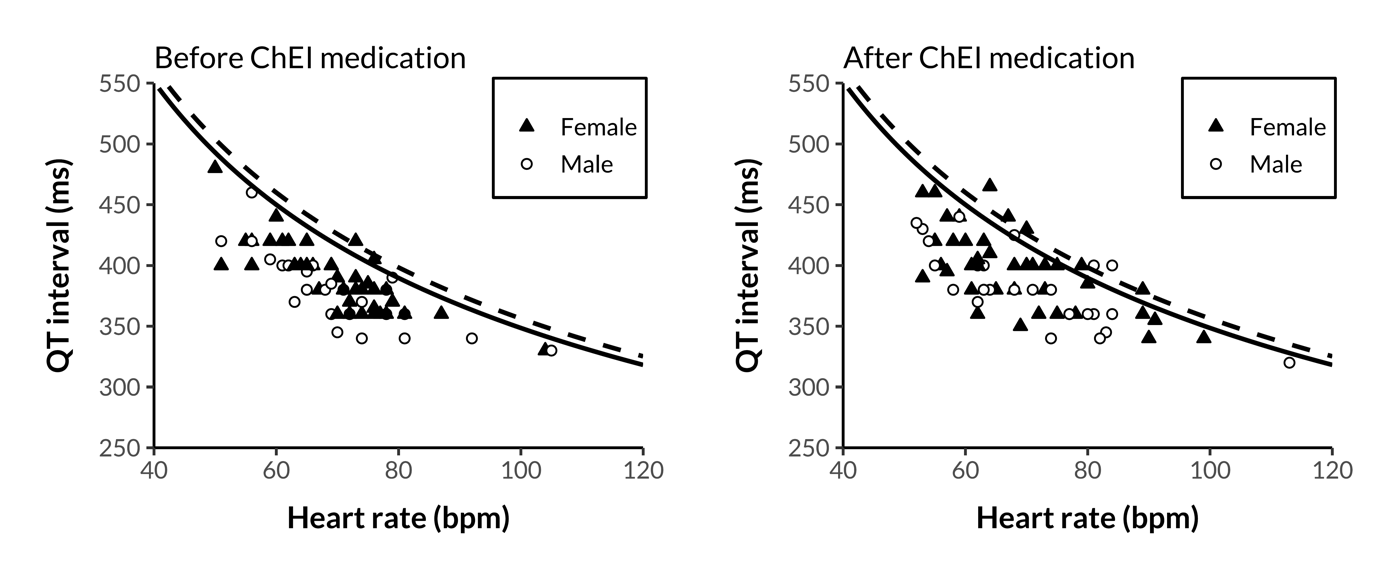


**Figure S3.** Scatter plots of QT interval by heart rate before and during ChEI medication in a subpopulation comprising only patients with manual QT measurements available for all six leads. The solid line represents the threshold for a pathological corrected QT interval in males (≥ 450 ms), while the dashed line indicates the threshold for females (≥ 460 ms). ChEI, cholinesterase inhibitor; bpm, beats per minute; ms, milliseconds
